# Risk factors for prolonged length of stay after first single-level lumbar microdiscectomy

**DOI:** 10.1007/s00701-024-05972-9

**Published:** 2024-02-13

**Authors:** Leonard Ritter, Adrian Liebert, Thomas Eibl, Barbara Schmid, Hans-Herbert Steiner, Ghassan Kerry

**Affiliations:** 1 Department of Neurosurgery, Paracelsus Medical University, Breslauer Str. 201, 90471 Nuremberg, Bavaria Germany; 2 Department of Neurology, Paracelsus Medical University, Breslauer Str. 201, 90471 Nuremberg, Bavaria Germany

**Keywords:** Lumbar microdiscectomy, Risk factors, Length of stay, Preoperative immobility, Lumbar herniated disc

## Abstract

**Objective:**

The objective is to identify risk factors that potentially prolong the hospital stay in patients after undergoing first single-level open lumbar microdiscectomy.

**Methods:**

A retrospective single-centre study was conducted. Demographic data, medical records, intraoperative course, and imaging studies were analysed. The outcome measure was defined by the number of days stayed after the operation. A prolonged length of stay (LOS) stay was defined as a minimum of one additional day beyond the median hospital stay in our patient collective. Bivariate analysis and multiple stepwise regression were used to identify independent factors related to the prolonged hospital stay.

**Results:**

Two hundred consecutive patients who underwent first lumbar microdiscectomy between 2018 and 2022 at our clinic were included in this study. Statistical analysis of factors potentially prolonging postoperative hospital stay was done for a total of 24 factors, seven of them were significantly related to prolonged LOS in bivariate analysis. Sex (*p* = 0.002, median 5 vs. 4 days for females vs. males) and age (*r*_*s*_ = 0.35, *p* ≤ 0.001, *N* = 200) were identified among the examined demographic factors. Regarding preoperative physical status, preoperative immobility reached statistical significance (*p* ≤ 0.001, median 5 vs. 4 days). Diabetes mellitus (*p* = 0.043, median 5 vs. 4 days), anticoagulation and/or antiplatelet agents (*p* = 0.045, median 5 vs. 4 days), and postoperative narcotic consumption (*p* ≤ 0.001, median 5 vs. 4 days) as comorbidities were associated with a prolonged hospital stay. Performance of nucleotomy (*p* = 0.023, median 5 vs. 4 days) was a significant intraoperative factor. After linear stepwise multivariable regression, only preoperative immobility (*p* ≤ 0.001) was identified as independent risk factors for prolonged length of postoperative hospital stay.

**Conclusion:**

Our study identified preoperative immobility as a significant predictor of prolonged hospital stay, highlighting its value in preoperative assessments and as a tool to pinpoint at-risk patients. Prospective clinical trials with detailed assessment of mobility, including grading, need to be done to verify our results.

## Introduction

While other surgical techniques have been gaining importance in recent years, lumbar microdiscectomy is still seen as the standard surgical procedure for the treatment of lumbar disc herniation in patients with relevant pain symptoms and neurological deficits that are refractory to conservative management [14].

It is one of the most common spinal procedures and thus accounts for a significant portion of the enormously high annual costs borne by the health care systems [16], with the USA alone spending nearly $50 billion annually on treating spinal issues [6, 10]. Also, hospital expenditures often have increased, as evidenced by Germany’s federal statistical office showing a 23.5% rise in patient costs from 2014 to 2019. Identifying factors that extend post-surgery hospital stay is therefore crucial for efficient and cost-effective patient care, as prolonged stays strain resources and reduce surgical capacities [14].

Prolonged hospitalization after surgery not only burdens healthcare systems but also heightens patients’ risks of postoperative complications, like deep vein thrombosis or hospital-acquired infections [4, 12].

The aim of this study was therefore to identify potential risk factors for prolonged inpatient stay after first single-level lumbar microdiscectomy. Currently, several studies are reported in the literature that analysed long hospital stay in other cohorts e.g., patients undergoing minimally invasive lumbar spine surgery (MIS), lumbar decompression surgery, or transforaminal lumbar interbody fusion [11, 17, 20]. However, to our knowledge, no study has yet been conducted that aimed specifically at patients after first single-level lumbar microdiscectomy.

## Methods

### Inclusion and exclusion criteria

Data of patients who underwent first single-level lumbar microdiscectomy between 2018 and 2022 were retrospectively reviewed. Inclusion criteria were a minimal age of 18 years and a symptomatic lumbar disc herniation confirmed radiographically by MRI. Patients with a history of previous lumbar spine surgery, as well as previous traumatic and recurrent lumbar disc herniations, were excluded from this study.

### Data collection

Demographic data, medical records, intraoperative course, and imaging studies were retrospectively analysed. Since the primary outcome of our study relates to the patient’s discharge date and no further observations beyond that date were included in the study, no follow-up or further data were recorded. Narcotic consumption was measured in oral morphine equivalents (OME). Immobility was subjectively attributed to the patients by the physician who made the initial contact based on the ability to stand up and walk without assistance. The factors “spinal stenosis” and “additional lumbar disc herniation (LDH)” refer to pathologies at other levels than the one it was operated on. A complete listing of all factors is presented in Table 1. The patients’ health status was determined by the American Society of Anaesthesiologists (ASA) risk classification, and the Charlson Comorbidity Index (CCI index) was collected and analysed separately from the other risk factors (Table 2).

**Table 1:** Descriptive statistics and bivariate analysis

Factors	Categories	(*n*)	(%)	Mean LOS (Days)	*p*-value	*r*
Sex	Female Male	**84** **116**	**42** **58**	**5** **4**	**0.002**	**0.22**
Age	** > **65 ** ≤ **65	**47** **153**	**23.5** **76.5**	**5** **4**	**0.002**	**0.33**
Preoperative immobility	No Yes	**96** **95**	**50.3** **49.7**	**5** **4**	** < 0.001**	**0.54**
Paresis	No Yes	88 95	48.1 51.9	4 4	0.29	
Pain	No Yes	5 195	2.5 97.5	4 4	0.590	
Hypoesthesia	No Yes	36 142	20.2 79.8	4 4	0.428	
Loss of reflexes	No Yes	82 91	47.4 52.6	4 4	0.252	
Hypertension	No Yes	117 83	58.5 41.5	4 4	0.063	
DM type 2	No Yes	**174** **26**	**87** **13**	**4** **5**	**0.043**	**0.15**
Smoking	No Yes	183 17	91.5 8.5	4 4	0.058	
Spinal stenosis	No Yes	191 9	95.5 4.5	4 4	0.084	
Additional LDH	No Yes	193 7	98.5 1.5	4 4	0.855	
Depression	No Yes	184 16	928	4 4	0.063	
Hypothyroidism	No Yes	178 22	89 11	4 4	0.448	
Hypercholesterinaemia	No Yes	183 17	91.5 8.5	4 4	0.505	
Postoperative narcotic consumption	No Yes	**116** **84**	**58** **42**	**4** **5**	** < 0.001**	**0.39**
Anticoagulation/antiplatelet agents	No Yes	**179** **21**	**89.5** **10.5**	**4** **5**	**0.045**	**0.14**
Drainage	No Yes	79 121	39.5 60.5	4 4	0.677	
Duration of pain	≤ 1 week ≤ 1 month ≤ 3 months > 3 months	35 76 38 27	19.9 43.2 21.6 15.3	4 4 4 4	0.749	
Duration of surgery	≤ 90 min > 90–120 min > 120–180 min > 180 min	95 46 44 14	47.7 23.1 22.1 7	4 4 4 4	0.907	
Nucleotomy	No Yes	**18** **182**	**9** **91**	**4** **5**	**0.023**	**0.16**
Laminotomy	No Yes	164 36	82 18	4 4	0.155	
Hemilaminectomy	No Yes	169 31	84.5 1.5	4 4	0.402	
Partial arthrectomy	No Yes	182 18	91 9	4 4	0.893	

The boldface indicates statistical significance. The *p*-value was calculated by using Mann–Whitney *U*-test for dichotomic variables and Kruskal–Wallis test for categorical variables; ***r*** indicates the correlation coefficient

*LDH* lumbar disc herniation, *n* patient number, *DM* diabetes mellitus

**Table 2:** Health status

ASA — median value for patients with increased LOS ± IQR	2 ± 0	*p*-value 0.67*
ASA — median value for patients with increased LOS ± IQR	2 ± 0	
CCI — mean value for patients with normal LOS ± SD	0.53 ± 1.25	*p*-value 0.52*
CCI — mean value for patients with increased LOS ± SD	0.64 ± 1.14	

*ASA* American Society of Anaesthesiologists risk classification, *CCI* Charlson Comorbidity Index

*Mann–Whitney *U*-test

### Outcome assessment

Significant factors were identified as such, if their presence increased the patients’ median length of stay (LOS) by at least 1 day compared to the mean of nonaffected patients. For our study, LOS referred exclusively to the postoperative number of days the patients stayed in hospital.

The readiness to be discharged from the hospital was judged by the assigned surgeons based on the ability of voluntary bladder control, compensated pain symptoms, and sufficient mobility.

### Statistical analysis

Statistical analysis was carried out using the IBM SPSS® Version 27 for Windows 10 (IBM Corp. Released 2020. IBM SPSS Statistics for Windows, Version 27.0. Armonk, NY: IBM Corp). Metric variables are presented as means and standard deviations (± SD) and medians with interquartile range (± IQR). Categorial variables are presented as number (*n*) and percentage (%). Bivariate statistical analysis was performed to evaluate the relation of the potential risk factors and prolonged hospital by using the Mann–Whitney *U*-test and Kruskal–Wallis test. To identify the most probable cause of bias in our statistics, the mean age of all factors that significantly influenced LOS in bivariate analysis was compared using Student’s *t*-tests. Correlation analyses were performed using Spearman’s correlation with Spearman’s Rho (*r*_*s*_) for all significant factors in the bivariate analysis. Independent risk factors were identified by using a multivariable linear stepwise regression model with all factors being initially included. Afterwards, the factors were excluded step by step until only those being significant remained. A *p*-value < 0.05 was considered significant in two-tailed testing.

## Treatment protocol

### Decision to treatment

The decision for operative treatment of lumbar disc herniations in our department is based on the correlation of clinical presentation and imaging. Patients with paresis or conus-cauda-syndrome were primarily operated, other cases only after failure of conservative treatment.

### Surgical procedure

All patients underwent single-level lumbar microdiscectomy. Small variations in the surgical management might have occurred as the patients included in the study were operated on by different surgeons. The primary goal of each operation was pressure relieve of the affected nerve root by removing the herniated disc fragment. If a rupture of the fibrous ring was found intraoperatively, a nucleotomy was performed as well to minimize the risk of recurrence. If the nerve root appeared to be still constricted on subsequent palpation, decompression of the root was performed as a final step.

The patients were operated on by different surgeons. However, each operation was performed by a specialist or under the supervision of a specialist.

### Management of dural tear

In the event of an intraoperative dural tear, the affected site is normally closed with a combination of suturing and subsequent taping. If possible, a dural suture is performed first and after that the dural tear is sealed with TachoSil®, a patch consisting of collagen, fibrin, and thrombin. Postoperatively, patients are kept supine in bed rest for 3 days. If they are symptom-free, they are allowed to elevate the head of the bed at 30°. After 3 days, the first careful assisted mobilization under monitoring for signs of CSF leakage takes place. If tolerated without symptoms, patients are allowed to ambulate as they want. In case of persistent headache or serous leakage from the wound, further diagnostics are conducted (MRI and beta (*β*)-2 transferrin testing). In patients with positive diagnostic results, the indication for a reoperation was given.

## Results

### Patient cohort

A total of 200 patients who were operated on at our clinic between 2018 and 2022 were included in this study. The mean postoperative hospital stay was 4.73 ± 2.22 days (median 4 ± 0, min 1, max 16). Among them, 84 (42%) were female and 116 (58%) were male. 119 (59.5%) patients had a normal LOS, and 81 (40.5%) had an increased LOS. All other factors that were analysed are presented in Table 1.

### Health status

To create comparability regarding the health status, the median ASA risk classification as well as the mean CCI values of both patient groups were compared before further bivariate and multivariable analysis. We did not include ASA and CCI among the other risk factors but analysed them separately. The reason for this is that the health status of the patients has an enormous influence on the length of hospital stay; a different health status of the two groups would thus generate a fundamental bias for the further analysis.

In our study, there was no statistical difference in the health status, concerning the ASA and the CCI Index, between patients with a normal LOS and patients with a prolonged LOS (Table 2).

### Results of bivariate analysis

Statistical analysis of factors potentially prolonging postoperative hospital stay was done for a total of 24 factors. The results of the bivariate analysis, the median LOS of all factors, and subgroups as well as the correlation coefficients can be seen in Table 1.

Sex (*p* = 0.002) was a significant demographic factor. Mean age for men was 51.6 ± 13.9 (median 51.5 ± 8.5) in comparison to the mean age for women 55.4 ± 16.1 (median 53.5 ± 16.3). Student’s *t*-test trended towards a significance in the age distribution (*p* = 0.10, CI =  − 7.67–0.78). Another demographic factor that was significant was the age of the overall population (*p* = 0.002).

**Fig. 1:**
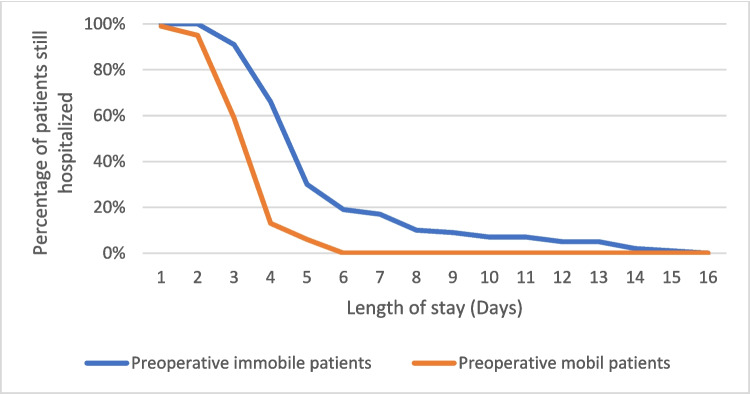
Time course of LOS for preoperatively immobile vs. mobile patients

Regarding preoperative physical status, only preoperative immobility reached statistical significance (*p* ≤ 0.001). Figure 1 shows the distribution of hospital discharges of preoperative mobile and immobile patients in relation to the LOS. The figure indicates a shift towards a delayed time of discharge for immobile patients. Thus, no patient who was mobile preoperatively left the hospital after the sixth day and no patient that was immobile preoperatively was discharged before the third postoperative day.

The intake of anticoagulation and/or antiplatelet agents (*p* = 0.045) was significantly related to LOS. The distribution of mean age and Student’s *t*-test showed that patients under those medications were considerably older than those who did not take anticoagulation and/or antiplatelet agents (mean 70.86 ± 10.9 vs. 51.1 ± 13.9, *p* < 0.001).

Diabetes mellitus (*p* = 0.043) as a comorbidity and postoperative narcotic consumption (p ≤ 0.001) were also associated with a prolonged LOS.

Performance of nucleotomy (*p* = 0.023) was a significant intraoperative factor.

### Results of multivariate analysis

For the multiple stepwise regression analysis, the presence of spinal stenosis and the performance of partial arthrectomy had to be excluded from the model as they lacked linear correlation. From the remaining 22 characteristics, only preoperative immobility (*p* ≤ 0.001, CI = 0.08–2.47) was shown to be an independent risk factor.

In our model, 40.2% (*R*^2^ = 0.402) of the scattering in the risk of prolonged LOS after first lumbar microdiscectomy could be explained, resulting in a moderate effect size according to Cohen (1992) (Table 3).

**Table 3:** Independent risk factors

Factor	*β*	SE	*p*-value	95% CI
Preoperative mobility	1.62	0.42	< 0.001	0.76–2.47

*p*-value was calculated by using multiple linear stepwise regression with sex, age, hypertension, diabetes mellitus, smoking, additional LDH, depression, hypothyroidism, hypercholesterinaemia, postoperative narcotic consumption, anticoagulation/antiplatelet agents, duration of pain, preoperative immobility, duration of surgery, nucleotomy, laminotomy, hemilaminectomy, dural tear, drainage, paresis, pain, hypesthesia, and loss of reflexes being initially included into the model

*β* standardized coefficient beta, *SE* standard error, *CI* confidence interval

## Discussion

Our study had the aim to identify factors that increase patients’ LOS after undergoing microdiscectomy for lumbar disc herniation. A total of 24 factors were tested, seven of which showed a significant effect in the bivariate analysis. After multivariable regression analysis, preoperative immobility remained as an independent risk factor.

### Demographic factors

Sex was a potential risk factor as women had a prolonged mean LOS of 1 day. However, the result is questionable in terms of reliability, since the mean age of men and women differs considerably, although not yet statistically significant.

In our study, age over 65 years was significantly correlated with LOS. This result reproduced and supported previous studies that presented a similar finding [7, 17, 22]. Khanaan et al. explained the correlation as a consequence of a greater number of comorbidities and a higher rate of complications after surgery [17]. However, age was not identified as an independent risk factor in the multiple regression presumably because of the restricted correlation and limited effect on LOS resulting in a modest percentage of variation in our regression model.

### Comorbidities

Regarding comorbidities, the only significant risk factor in bivariate analysis of our study was diabetes mellitus with diabetic patients having a median prolonged stay of 1 day. This finding is consistent with previous studies dealing with lumbar spine surgery [2, 13, 20]. Maloney et al. demonstrated an approximately 1.4-fold increase (1.9 vs 1.4 days) in LOS in a diabetic population undergoing open lumbar microdiscectomy [2]. Furthermore, Guzman et. al showed that controlled diabetes increased the LOS after degenerative lumbar spine surgery only by half a day (1.1-fold), while patients with uncontrolled diabetes mellitus had an increase of about 2.4 days (1.7-fold). This finding demonstrates the importance of glycaemic control in the perioperative period. That is especially relevant in patients with certain oral antidiabetics, which may have to be discontinued and substituted by insulin perioperatively.

### Medication

The intake of anticoagulation and/or antiplatelet medication was significantly related to LOS; however, the factor seemed to be more of a confounder than an independent predictor for LOS as patients taking anticoagulation and/or antiplatelet medication were significantly older than those who did not.

The results of our study suggest that a postoperative need for opioids is associated with a prolonged LOS. A similar observation was made in other studies in patients with a high postoperative Visual Analogue Scale (VAS) [3]. However, identifying pain as an independent risk factor for prolonged LOS is quite difficult due to the subjective individual experience of pain on the one hand and the various potential confounding factors like an emotional response or anxiety on the other hand [9]. Furthermore, the prevalence of patients with chronic pain and with increased narcotic demand is especially high among those with spinal disorders [5]. Under consideration of possible aggravating factors that may intensify the patients’ experienced pain after surgery, adequate pain management is imperative to improve the functional outcome after surgery. As inadequately treated postsurgical pain contributes to longer hospital stays, slower progress in ambulation and development of chronicity of functional deficits [8]. Overall, the need for opioids after an operation appears to be more useful as an indirect measure of postoperative pain than as a predictive factor in itself.

### Nucleotomy

Comparing surgical techniques, performance of nucleotomy plus sequestrectomy increased the median LOS by one day in contrast to simple sequestrectomy. A previous meta-analysis by Huang et. al from 2015 identified three studies comparing LOS after these two surgical techniques [1]. LOS in these studies ranged from 0.9 to 6.4 days in the sequestrectomy group and from 1.17 to 6.94 days in the nucleotomy group. While all three studies concluded shorter LOS after sequestrectomy, the difference was not statistically significant. Though, it can be concluded that while there might be some difference between the two surgical techniques, the correlation to LOS was modest (*r* = 0.16). Thus, our data supports the findings of previous studies that the choice between the two surgical techniques has no statistically relevant effect on the length of postoperative hospital stay.

### Preoperative physical status

In our study, preoperative immobility prolonged LOS by 1 day and had also the strongest positive correlation with prolonged LOS (*r* > 0.5) of all tested factors. Regarding potential bias, there was no significant difference in the distribution of other clinical examination factors (pain, paresis, hypesthesia, or loss of reflexes) between the two groups.

The reasons that may influence preoperative immobility or decreased physical function are widespread. Among these are enhanced pain sensation, psychological, and psychiatric pre-existing conditions [19]. In addition, Bernstein et al. showed that socioeconomic disadvantage can have a negative impact on physical function during the initial clinical presentation in lumbar disc herniations (*p* ≤ 0.001) [21].

The strength of the survey of preoperative immobility as a predictor therefore results from the fact that it indirectly combines many other factors to one single factor. Apart from that, the assessment of preoperative immobility to predict prolonged LOS is easy and quick, as it does not need additional information to calculate the result. Our study has shown that the determination of preoperative immobility is particularly accurate in identifying patients who had a significantly prolonged LOS. All patients with a postoperative LOS of more than 6 days were classified with preoperative immobility and could be identified as at-risk patients for prolonged LOS during the initial assessment of the physical status.

Patients whose LOS is well above average are particularly at risk of complications and require significantly higher level of resources. The fact that the assessment of preoperative immobility was able to precisely identify these patients increases the clinical benefit of this factor. By using preoperative immobility as predictor, healthcare providers can tailor interventions and develop targeted strategies, such as better conservative pain management or periradicular infiltrations, to optimize postoperative recovery.

Limitations of the results arise, because in its current binominal form and therefore without the possibility of gradations, the factor loses informational content as it cannot determine the degree of severity in comparison to established clinical scores. Due to its limitations, the preoperative immobility as a predictor needs to be graded and be implemented as a score to make this factor comparable in future studies.

Beside preoperative immobility, no other factor of the initial physical status at the time of admission was significantly associated with LOS. This finding is consistent with other studies that were using different outcome measures like the Short Form (36) Health Survey (SF-36), Visual Analogue Scale (VAS), or Oswestry Disability Index (ODS) to predict outcome after lumbar microdiscectomy [15, 18].

## Limitations of this study

Our present study is inherently limited by its retrospective observational design. Also, the possible reasons for an extended LOS can be manifold and may include reasons that cannot simply be recorded as factors. It was therefore probably not possible to eliminate all potential bias in our study.

However, our data contribute to the increasing effort in defining the factors which influence LOS in this patient population.

## Conclusion

In our study, we could identify multiple factors which seem to influence LOS. Among them, preoperative immobility demonstrated as the most important and independent risk factor for prolonged hospitalization. This finding underscores the importance of preoperative assessments and demonstrates the usage of preoperative immobility as a valuable predictor to identify patients at risk for prolonged LOS.

By identifying this specific risk factor, healthcare providers can tailor interventions and develop targeted strategies, such as focused and intensified conservative pain management or periradicular therapy, that might help to optimize postoperative recovery.

Prospective clinical trials with detailed assessment of mobility, including grading, need to be done to verify our results.

## Data Availability

The datasets used and analysed during the current study are available from the corresponding author on reasonable request.

## Abbreviations

ASA: American Society of Anaesthesiologists risk classification
CCI: Charlson Comorbidity Index
IQR: Interquartile range
LDH: Lumbar disc herniation
LOS: Length of stay
MIS: Minimally invasive lumbar spine surgery
ODS: Oswestry Disability Index
OME: Oral morphine equivalent
SF-36: Short Form (36) Health Survey
SD: Standard deviation
VAS: Visual Analogue Scale
